# Toxicity of rectal preparation strategies in ultra-hypofractionated SBRT for prostate cancer

**DOI:** 10.1016/j.tipsro.2026.100405

**Published:** 2026-04-19

**Authors:** R. van der Walle, F.H.E. Staal, J. Janssen, J.A. Langendijk, C.L. Brouwer, S. Aluwini

**Affiliations:** University Medical Center Groningen, Dept. Of Radiation Oncology, Groningen, The Netherlands

**Keywords:** Prostate cancer, Stereotactic body radiotherapy (SBRT), Rectal preparation, Gastrointestinal toxicity

## Abstract

**Background and purpose:**

Ultra-hypofractionated radiotherapy using stereotactic body radiotherapy (SBRT) in five fractions is standard care for patients with low- or intermediate-risk prostate cancer. Rectal preparation aims for consistent rectal filling to improve dose accuracy and reduce toxicity, however, current evidence is inconsistent and based on conventional fractionation. This study compares four rectal preparation strategies in terms of rectal volume, dose delivery, and acute gastrointestinal toxicity.

**Materials and methods:**

A total of 56 patients were allocated to four groups with different rectal preparation strategies: (1) no preparation (N = 14); (2) laxatives and a high‑fiber diet before planning CT and during treatment (N = 14); (3) laxatives before planning CT, without laxatives during treatment and without diet (N = 14); and (4) laxatives before planning CT and during treatment, without diet (N = 14). In total, 280 pre‑treatment cone‑beam CT scans were analyzed. Rectal volumes and dosimetric parameters were extracted and compared across groups. Both patient‑reported and physician‑reported gastrointestinal toxicity were evaluated.

**Results:**

Groups receiving extensive (laxatives and diet) or no preparation showed slightly greater rectal volume stability compared to groups receiving laxatives or laxatives only before planning CT (p = 0.001). Most dose parameters were comparable between groups. The group that received laxatives only before planning CT showed higher absolute rates of patient and physician reported gastrointestinal toxicity, although these differences were not statistically significant.

**Conclusion:**

None of all rectal preparation strategies consistently improved rectal volume stability, provided dosimetric benefit, or reduced gastrointestinal toxicity compared with no preparation. These findings do not support a meaningful benefit of routine use of rectal preparation in the context of modern daily CBCT-guided and adaptive radiotherapy workflows. Future research should prioritize patient-centred, less invasive approaches as the current evidence does not justify the routine use of rectal preparation.

## Highlights

• One of the first studies reviewing rectal preparation in SBRT setting.
• Value of incorporating patient and physician reported clinical outcomes.
• Extensive rectal preparation with diet and laxatives does not translate into consistent clinical benefit.
• Based on current evidence, routine application of rectal preparation is not supported.

## Introduction

Ultra-hypofractionated radiotherapy using stereotactic body radiotherapy (SBRT) has emerged as a promising approach for prostate cancer (PCa), offering shorter treatment schedules of only five fractions. Phase III randomized controlled trials (RCTs) have demonstrated that ultra-hypofractionated SBRT achieves non-inferior failure-free survival outcomes and similar rates of gastrointestinal (GI) toxicity in patients with low and favourable intermediate-risk PCa [1], [2], [3], [4]. Consequently, ultra-hypofractionated SBRT has become the standard of care for these patients.

SBRT requires precise targeting, as high fractional doses in few sessions increase the risk of geographic miss and organ-at-risk (OAR) exposure. Daily variations in rectal filling can affect prostate position, shape, and therefore dose delivery [5]. As a result, rectal preparation has been proposed to improve treatment reproducibility, enhance dosimetric accuracy, and reduce rectal toxicity.

Rectal preparation strategies range from dietary adjustments, laxatives, and antiflatulent agents to invasive interventions such as enemas or rectal spacers. However, evidence remains inconsistent and heterogeneous, and is mostly derived from non-SBRT settings [6]. Only a few studies have specifically assessed preparation in SBRT, despite its greater sensitivity to anatomical variation.

In the context of conventional (non-SBRT) prostate radiotherapy, evidence regarding rectal preparation is likewise restricted and inconsistent. One RCT found the osmotic laxative Movicolon more effective in reducing rectal gas than the bulking laxative Metamucil [7], whereas another study showed no benefit of antiflatulent use [8]. Notably, most of these studies evaluated surrogate endpoints, such as rectal volume or gas presence on imaging, rather than clinically relevant and patient-centred outcomes, such as toxicity, thereby limiting the clinical applicability of their findings.

Hence, there is currently no clear conclusion on the optimal rectal preparation strategy for ultra-hypofractionated SBRT. To address this gap, the aim of this study was to evaluate the impact of four different rectal preparation strategies on rectal volume stability, dose delivery, and acute GI toxicity in patients undergoing SBRT for PCa.

## Materials and methods

After institutional ethics board approval, a total of 56 patients with prostate cancer undergoing ultra-hypofractionated radiotherapy between February 2021 and November 2024 were consecutively selected based on their rectal preparation regimen. During this time, routine clinical rectal preparation strategies progressively evolved in response to clinical observations, patient feedback, and growing experience during planning and treatment delivery. For each strategy, fourteen patients were retrospectively included. As a result, the following four distinct rectal preparation strategies were implemented in successive cohorts:

a. No preparation: no laxatives and no diet (N=14).

b. Laxatives and high-fiber diet before planning CT and during treatment (N=14).

c. Laxatives before planning CT, no laxatives during treatment, without diet (N=14).

d. Laxatives before planning CT and during treatment, without diet (N=14).

Eligibility criteria included low or intermediate risk prostate cancer, defined as following: low risk (PSA ≤10 ng/mL, and GSS ≤6, cT1-2a) and intermediate risk (PSA 10-20 ng/mL and/or GSS 7 and/or cT2b).

### Treatment

Patients received 5 fractions of 7.25 Gy delivered every other day using VMAT in two arcs. For low-risk patients, the clinical target volume (CTV) included the prostate only, while for intermediate-risk patients, the CTV encompassed the base of the seminal vesicles (1 cm). A 5 mm planning target volume (PTV) margin was applied. OAR dose constraints are detailed in Appendix A. Adequate PTV coverage was defined as 95% of the PTV receiving at least 99% of the prescribed dose. All patients received gold fiducial markers. Daily treatment verification was performed using pre-treatment cone-beam CT (CBCT) registered to planning-CT based on fiducial markers. An additional intra-treatment CBCT was acquired after the first VMAT arc and matched similarly; any positional deviation of ≥1 mm was corrected immediately. Image registration was performed using Elekta XVI (Stockholm, Sweden).

### CBCT imaging, rectal volume and dosimetric parameters

A total of 280 pre-treatment CBCT images (five per patient, one per fraction) were imported into Mirada RTx (Mirada Medical Ltd, Oxford) and registered to the corresponding planning-CTs using the fiducial marker-based verification protocol. The bladder and rectum were delineated on all CBCTs by three observers. The anorectal wall and bladder wall were defined as the outermost 3mm of each organ. All contours were transformed to the planning CT to derive dose parameters for each (pre)treatment fraction.

Rectal volumes during treatment were compared to the planning CT volumes across the four preparation groups. Protocol-defined dose parameters for anorectum (V30Gy, V20Gy, Maximum), anorectal wall (V28Gy, V18,5Gy, Maximum) and anal canal (Dmean, V25cc, Maximum) were assessed and compared with the corresponding planning-CT doses. To account for baseline intergroup differences in rectal volume, percentage volume change relative to the planning CT were used for group comparisons. Percentage change from the planning CT was calculated for both rectal volume and dosimetric parameters by comparing each fraction to the planning value: (fraction value – planning value) / planning value × 100%.

### Intrafractional motion

Intrafractional motion was assessed using clinical CBCT matching results. An intrafractional CBCT was performed between the 2 treatment arcs to verify patient positioning. When displacement of ≥1 mm in any axis was observed, the patient position was corrected. The frequency of intrafraction corrections per treatment fraction (5 fractions per patient) was collected for each rectal preparation group.

### Toxicity

Patients completed the modified Radiation Therapy Oncology Group / European Organisation for Research and Treatment of Cancer (RTOG/EORTC) Acute Radiation Morbidity Criteria questionnaires at baseline, end of treatment and 3 months after treatment as part of our standardized prospective follow-up program. Patient-reported GI toxicity derived from these questionnaires was classified according to the RTOG Acute Radiation Morbidity Criteria, consistent with prior studies [9], [10]. Patients were included in this analysis if they have completed both the baseline and at least one follow-up questionnaire (end treatment or 3-month after treatment).

Physician reported toxicity was retrospectively scored based on clinical patient records, as per the Common Terminology Criteria for Adverse Events (CTCAE version 5.0). To assess the impact of laxative use on gastrointestinal toxicity, we extracted and scored toxicity events that were explicitly attributed to laxatives in the physician’s clinical notes.

### Statistics

Group comparisons were performed using one-way ANOVA for continuous variables with a normal distribution and the chi-square test for categorical variables. A two-sided p-value of <0.05 was considered statistically significant.

Within each group, the Wilcoxon signed-rank test was used to compare median rectal volumes and dosimetric parameters across treatment fractions relative to the planning CT. Kruskal-Wallis was used for between-group comparisons of the percent deviations from planned to delivered volume and doses. Post hoc pairwise comparisons were performed using Dunn test with Bonferroni correction (p < 0.05). Proportions of fractions requiring a re-match due to intrafractional displacement were compared between groups using chi-square test with pairwise post hoc comparisons and Bonferroni correction.

Toxicity outcomes were primarily analysed descriptively due to small sample sizes. Fisher’s exact test was applied to compare absolute toxicity rates at specific time points. For binary patient-reported grade ≥2 GI toxicity, a generalized linear mixed model (GLMM) accounting for repeated measures was used to assess changes over time and between-group differences. Physician-reported toxicity, classified as an ordinal outcome based on CTCAE scores, was analysed using a cumulative link mixed model (CLMM) accounting for repeated measures. All statistical analyses were performed using R version 4.4.2 (R Foundation for Statistical Computing, Vienna, Austria).

### Ethical considerations

This study was approved by the Clinical Trial Committee (CTC) of the University Medical Center Groningen (UMCG) (protocol number: 20265). All patients provided written informed consent for the use of their data as part of our Standardized Follow-Up-program (clinicaltrial.gov NCT02435576). All data were anonymized prior to analysis and handled in accordance with applicable data protection regulations. The study was conducted in accordance with the principles of the Declaration of Helsinki.

## Results

In total, 280 pre-treatment CBCT scans of 56 patients were analysed. All CBCT scans were deemed of adequate quality for delineation and analysis. Baseline characteristics for each rectal preparation group are displayed in Table 1. No significant imbalances were observed except for ISUP grade.

**Table 1:** Baseline characteristics by preparation group

	**1: No preparation** N = 14^a^	**2: Diet + laxatives** N = 14^a^	**3: Laxatives only plan CT** N = 14^a^	**4: Laxatives** N = 14^a^	**p-value^2^**
**Age**	69.2 ± 5.9	73.2 ± 4.8	74.1 ± 5.3	73.6 ± 4.8	0.12
Initial PSA*	8.4 ± 3.8	9.5 ± 3.0	9.5 ± 3.0	10.5 ± 3.0	0.48
WHO-PS*					0.15
0	12 (86%)	11 (79%)	11 (79%)	7 (50%)	
1	2 (14%)	2 (14%)	3 (21%)	7 (50%)	
2	0 (0%)	1 (7.1%)	0 (0%)	0 (0%)	
ISUP*					0.03
1	0 (0.0%)	3 (21.4%)	0 (0.0%)	0 (0.0%)	
2	12 (85.7%)	7 (50.0%)	10 (71.4%)	6 (42.9%)	
3	2 (14.3%)	4 (28.6%)	4 (28.6%)	8 (57.1%)	
**Clinical T-stage**					0.36
1	9 (64%)	5 (36%)	9 (64%)	9 (64%)	
2	5 (36%)	9 (64%)	5 (36%)	5 (36%)	

*PSA: prostate-specific antigen; WHO-PS: World Health Organization performance status; ISUP: International Society of Urological Pathology.

a Mean ± SD; n (%) ^2^ P-values from one-way ANOVA or Fisher’s Exact test.

### Rectal volume

**Fig. 1:**
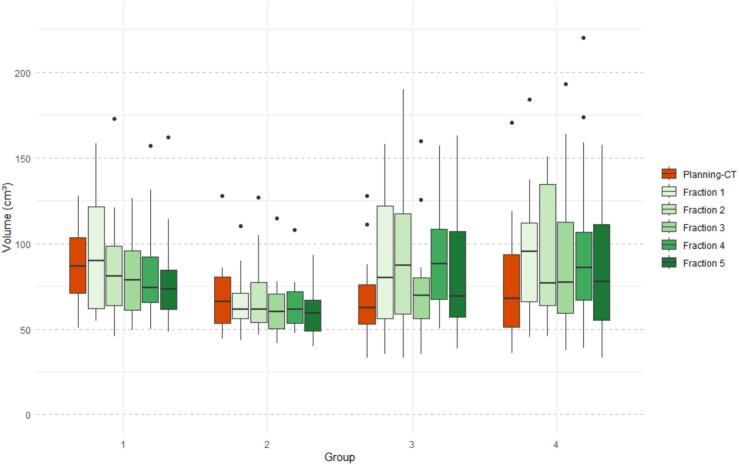
Median rectal volume per group at planning CT and during fractions 1–5. Group 1: No preparation; Group 2: Diet + laxatives; Group 3: Laxatives only planning-CT; Group 4: Laxatives.

Rectal volumes from planning CT and during treatment are shown in Table 2 and Fig. 1. Group 2 (diet + laxatives) showed the most stable median rectal volumes (66.0 vs 60.4 cm^3^). Group 1 (no preparation) had the highest median rectal volume at planning CT (86.9 cm^3^), which significantly decreased during treatment (73.7 cm^3^). In contrast, Groups 3 (laxatives only before planning CT) and 4 (laxatives) demonstrated a significant increase in median rectum volume during treatment compared to the planning CT. Intergroup analysis confirmed significantly better rectal volume stability in Groups 1 and 2 compared to Groups 3 and 4 (Kruskall Wallis, p<0.001). Detailed post hoc comparisons are reported in Appendix B.

**Table 2:** Median (IQR) rectal volumes on planning CT versus treatment fractions

	Planning-CT	Treatment	p value a	r (effect size)
Group 1: No preparation	86.9 (70.6 – 103.2)	73.7 (63.5 – 83.9)	0.02	-0.57
Group 2: Diet + laxatives	66 (52.4 – 79.5)	60.4 (56.8 – 63.9)	0.13	0
Group 3: Laxatives only planning-CT	62.3 (50.8 – 73.8)	76 (67.9 – 84.1)	0.02	0.43
Group 4: Laxatives	67.9 (46.8 – 89)	80 (68.6 – 91.3)	0.02	0.71

a Wilcoxon test for in group comparison of median volumes.

### Dosimetric parameters

The maximum dose to the anorectum and anorectal wall was significantly higher during treatment compared with planning-CT across all groups, although no significant differences were found between the groups.

In contrast, a significantly increase in anal canal V25 was observed in only Group 4 (laxatives) rising from 2.2 cc at planning to 3.2 cc during treatment. This increase was accompanied by a corresponding increase in the maximum anal canal dose, from 37.7 Gy to 38.5 Gy. Complete dosimetric data are provided in Appendix C, and adjusted post hoc results in Appendix D.

### Intrafractional motion

Among all four groups, displacement of ≥1 mm detected on the intrafraction CBCT requiring repositioning between the two treatment arcs, was least frequent in Group 2 (diet and laxatives), occurring in 62.7% of fractions, and most frequent in Group 3 (laxatives only planning-CT); occurring in 84.3% of fractions (p = 0.045), full data are reported in Appendix E and F.

### Toxicity

*Patient reported toxicity according to the RTOG*/*EORTC questionnaires*.

The mean questionnaire response rate was 64%. At end of treatment, the number of patients experiencing grade ≥2 GI toxicity ranged from 2 to 3 in each group. At 3 months, grade ≥2 toxicity had resolved in Group 1 and was reported by only 1 patient in Groups 2 and 4. In contrast, 3 patients in Group 3 continued to experience grade ≥2 symptoms. These differences were not statistically significant (p = 0.446, Fisher’s exact test). The most frequently reported symptoms were loose urge, cramping urge, and mucus discharge.

GLMM analysis showed no significant differences in toxicity between groups (all adjusted p > 0.05), but indicated a non-significant trend towards higher toxicity in Group 3 (laxatives prior to planning CT only), with an estimated rate of 22%, compared to 3–5% in the other groups (p > 0.28). Detailed scores are summarized in Table 3, with full results per symptom in Appendix G.

**Table 3:** Gastrointestinal (GI) toxicity per group and timepoint.

Grade 2: Patient reported (RTOG*)	**Baseline**	**Treatment**	**3 months**
*Group 1: No preparation*	0 (0%)	3 (27.3%)	0 (0%)
*Group 2: Diet + laxatives*	0 (0%)	2 (20%)	0 (0%)
*Group 3: Laxatives only planning CT*	0 (0%)	3 (50%)	3 (42.9%)
*Group 4: Laxatives*	0 (0%)	2 (28.6%)	1 (12.5%)

Grade 1: Physician reported (CTCAE*)			
*Group 1: No preparation*	1 (0%)	1 (7.1%)	2 (14.3%)
*Group 2: Diet + laxatives*	0 (0%)	7 (50.0%)	1 (7.1%)
*Group 3: Laxatives only planning CT*	2 (14.3%)	3 (21.4%)	3 (21.4%)
*Group 4: Laxatives*	1 (7.1%)	4 (28.6%)	1 (7.1%)

Grade 2: Physician reported (CTCAE*)			
*Group 1: No preparation*	0 (0%)	1 (7.1%)	0 (0%)
*Group 2: Diet + laxatives*	0 (0%)	0 (0%)	0 (0%)
*Group 3: Laxatives only planning CT*	0 (0%)	2 (14.3%)	0 (0%)
*Group 4: Laxatives*	0 (0%)	1 (7.1%)	0 (0%)

n (%) = number of patients with Grade ≥2 GI toxicity / number of evaluable patients at each time point.

* RTOG: Radiation Therapy Oncology Group; CTCAE: Common Terminology Criteria for Adverse Events.

*Physician reported; CTCAE*

**Fig. 2:**
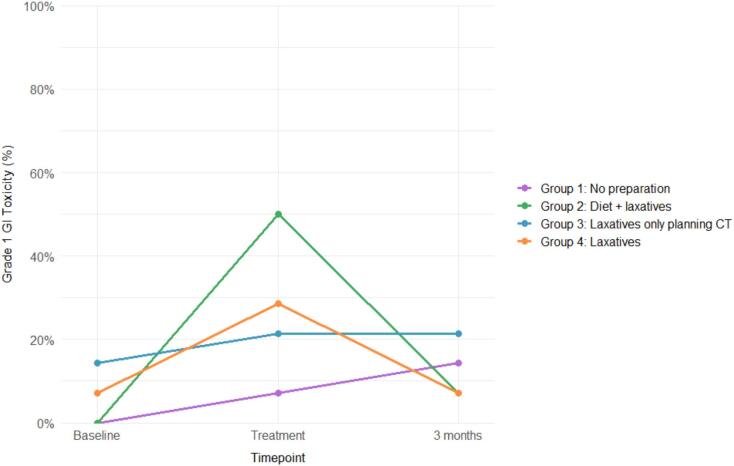
Physician reported Grade 1 gastrointestinal (GI) toxicity (CTCAE). CTCAE: Common Terminology Criteria for Adverse Event.

Physician-reported toxicity data were available for 96% across all timepoints. One patient in Group 2 developed a Grade 3 GI toxicity, presenting with a prostate abscess following fiducial implantation. Grade 1 GI toxicity occurred most often in Group 2 (50%) and least in Group 1 (7.1%), but differences were not significant (p = 0.114). Results are illustrated in Fig. 2. The overall incidence of Grade ≥2 CTCAE toxicity was 7.1%, without significant intergroup differences (p = 0.895).

CLMM analysis showed no significant inter-group differences in GI toxicity, though a relative increase in toxicity was estimated for Group 3 (laxative prior to planning CT).

GI toxicities and milder GI symptoms like diarrhoea and cramps were described in clinical records as being associated with laxative use in 36% of all patients in Group 2 and 29% in Group 4.

## Discussion

This retrospective analysis of prospectively collected data provides new insights into the clinical relevance of various rectal preparation strategies during ultra-hypofractionated prostate radiotherapy. No consistent improvement in rectal volume stability, dosimetric benefit, or reduction in GI toxicity was observed with any rectal preparation strategy compared to no preparation.

### Role of rectal preparation in the era of image guidance and adaptive radiotherapy

This study primarily focused on interfractional rectal variation, as rectal preparation is expected to mainly influence interfractional rather than intrafractional motion, consistent with previous studies [6], [11]. Using clinical CBCT data, a subset of intrafractional motion was captured, although this approach does not provide continuous tracking. Moreover, with the widespread use of daily CBCT-based image guidance, interfractional position changes can be corrected and anatomical changes can be directly monitored, thereby reducing the potential effect of rectal preparation. Also, in online CBCT-based adaptive radiotherapy, the treatment plan can be adapted to the anatomical shape of the day. Looking ahead, emerging techniques such as AI-driven anatomical tracking and Magnetic Resonance (MR)-guided adaptive radiotherapy hold promise for individualized treatment and enabling real-time correction of intrafraction motion, thereby enabling smaller margins and further minimizing treatment burden [12].

### Clinical implications of laxative use

Our findings suggest that laxative-based regimens are associated with GI side effects during treatment, including diarrhoea, abdominal discomfort and cramping, without clear benefit in rectal volume stability or grade ≥2 GI toxicity reduction. This aligns with previous reports and highlights the importance of patient compliance and comfort, often overlooked in earlier studies [6]. Recent prospective data demonstrate that patient compliance with intensive bowel preparation protocols is frequently suboptimal due to inconvenience and discomfort [13]. A patient-centred framework that integrates patient-reported outcomes (PROs), adherence and tolerability is therefore essential to appropriately evaluate the clinical feasibility and utility of rectal preparation strategies.

### Alternative strategies and focus on rectal gas

While intensive strategies such as laxative use may improve anatomical consistency, they are associated with side effects, patient discomfort, increased clinical resource use, and in case of more invasive strategies, such as spacers or rectal balloons, increased complication risks like bleeding or infection [14], [15], [16], [17]. Given the absence of demonstrable clinical benefit in our and prior studies, the routine use of the more invasive or intensive preparation strategies should be approached with caution. Therefore, less invasive strategies may warrant further investigation.

In particular, recent data suggest that rectal gas, rather than total rectal volume, may disproportionately impact prostate displacement and target coverage. Schaefer et al. demonstrated that a low FODMAP diet significantly reduced rectal gas and rectum volume (mean volume 64 cm3 vs. 71 cm3; p =0.02) [18], [19], [20]. Gas targeted strategies, like low-FODMAP diet or antifoaming agents, may be more effective and better tolerated than volume-focused regimens. Although our study did not include a diet-only group, the relatively more stable rectal volumes observed in Group 2 (diet + laxatives) could partially reflect a benefit from the dietary component. However, this hypothesis warrants further investigation.

### Guidelines perspectives

Current international guidelines, including those from ESTRO-ACROP, ASTRO, and NCCN, acknowledge the importance of rectal volume stability in prostate radiotherapy, but lack uniform recommendations regarding rectal preparation strategies [21], [22], [23]. Due to limited evidence supporting their efficacy, rectal preparation is left to local practice in selected cases. The guidelines do emphasize the use of image-guidance techniques and the importance of consistency, which aligns with our findings that show higher toxicity in the group where rectal preparation was applied inconsistently (only during preparation). Our findings add to the growing body of evidence questioning the necessity of routine preparation, like work of Alexander et al who do not support the need for rectal preparation when delivering contemporary IGRT [24]. This study also suggests that the omission of rectal preparation does not compromise dosimetric quality and may even reduce patient burden, supporting a more selective, individualized approach than currently implied by some clinical practices.

### Resource implications

Beyond clinical outcomes, rectal preparation strategies have important implications for workflow efficiency and resource utilization. Intensive regimens demand additional staff, time, logistical coordination, and may increase costs without proven benefit. In busy radiotherapy departments, simplified or omitted preparation protocols can streamline patient pathways, reduce treatment delays, and improve overall efficiency. Our data therefore support a pragmatic perspective: unless clear clinical advantages are demonstrated, rectal preparation should not be mandated, as its routine use may represent an unnecessary burden for both patients and healthcare systems.

### Strengths and limitations

Key strengths of this study include the simultaneous evaluation of multiple preparation strategies, and the incorporation of prospectively collected PROs. The integration of PROs with physician-reported toxicity is unique compared with previously published studies. The observed discrepancy between patient- and physician-reported outcomes emphasizes the added value of incorporating patient perspective, which is more sensitive to subjective symptoms, a finding consistent with prior literature [25]. Such comprehensive assessment is essential, as dosimetric or imaging parameters alone often underestimate the clinical impact of rectal preparation strategies.

Limitations include the small sample size within each group, which limits statistical power. The retrospective study design may have introduced selection bias. The questionnaire response rate of 64% could have led to non-response bias, although this was partly mitigated by physician-reported data available in 96% of cases. Target coverage of the prostate and oncologic outcomes were not captured. Although a significant difference in anal canal dosimetry between groups was observed, the magnitude was small and its clinical relevance remains uncertain.

In summary, none of all rectal preparation strategies did consistently improve rectal volume stability, show dosimetric benefit or reduce gastrointestinal toxicity compared to no preparation, based on both physician- and patient-reported outcomes. Therefore, this study does not support routine use of rectal preparation protocols in ultra-hypofractionated prostate radiotherapy. Without robust evidence supporting clinical benefits, invasive or intensive regimens, should be implemented cautiously, balancing benefits against side effects, patient burden, and resource use. Future (randomized) studies should prioritize patient-centred outcomes, including toxicity, comfort, adherence and quality of life while exploring innovative, less invasive strategies to define the optimal approach for daily clinical practice.

## Declaration of generative AI in scientific writing

During the preparation of this work the authors used Copilot and ChatGPT in order to improve readability and language. After using this tool, the authors reviewed and edited the content as needed and take full responsibility for the content of the publication.

## CRediT authorship contribution statement

**R. van der Walle:** Data curation, Formal analysis, Investigation, Methodology, Writing – original draft, Writing – review & editing. **F.H.E. Staal:** Conceptualization, Data curation, Methodology, Writing – review & editing. **J. Janssen:** Conceptualization, Data curation, Methodology, Writing – review & editing. **J.A. Langendijk:** Writing – review & editing. **C.L. Brouwer:** Writing – review & editing. **S. Aluwini:** Conceptualization, Methodology, Supervision, Writing – review & editing.

## Declaration of competing interest

The authors declare that they have no known competing financial interests or personal relationships that could have appeared to influence the work reported in this paper.

## Appendix A Supplementary data

Supplementary Data 1 Supplementary material including treatment planning constraints, post hoc analyses, detailed volumetric and dosimetric data, intrafractional motion, and symptom specification.
