# Supplementary material for: Toxicity of rectal preparation strategies in ultra-hypofractionated SBRT for prostate cancer
Source: Tech Innov Patient Support Radiat Oncol. 2026 Apr 19;38:100405. doi: 10.1016/j.tipsro.2026.100405 (PMC13127317; doi:10.1016/j.tipsro.2026.100405)
Supplement: Supplementary Data 1 — Supplementary material including treatment planning constraints, post hoc analyses, detailed volumetric and dosimetric data, intrafractional motion, and symptom specification. [file mmc1.docx]

**Toxicity of Rectal Preparation Strategies in Ultra-Hypofractionated SBRT for Prostate Cancer**

**APPENDICES**

| ***OAR*** | ***Constraint*** | ***% / cc / Gy*** |
| --- | --- | --- |
| *Anorectum* | V37.25  V30  V20 | < 2 cc  < 15%  < 30% |
| *Bladder* | Dmax  V37.5  V25 | 39.5 (on 0.1 cc)  < 5%  < 30% |
| *Anal canal* | Dmax  Dmean  V25 | < 38 Gy  < 19 Gy  < 10 cc |
| *Anorectal wall* | V28  V18.5  Dmax | < 15%  < 30%  < 38 Gy |
| *Femur left and right*  *(cumulative)* | V20  Dmax | < 2 cc  < 25 Gy |
| *Small intestine* | Dmax  V25 | < 35 Gy  < 10 cc |
| *Penile bulb* | Dmax | < 100% |
| *Skin* | Dmax | < 40% |

*Appendix A: Treatment planning constraints*

| **Group A** | **Group B** | **Z-score** | **p-value** | **Adjusted p-value** |
| --- | --- | --- | --- | --- |
| 1 | 2 | 0.510 | 0.610 | 1.00 |
| 1 | 3 | 3.175 | 0.001 | 0.007 |
| 1 | 4 | 3.268 | 0.001 | 0.007 |
| 2 | 3 | 2.665 | 0.008 | 0.023 |
| 2 | 4 | 2.758 | 0.006 | 0.023 |
| 3 | 4 | 0.093 | 0.926 | 1.000 |

*Appendix B: Post hoc comparison (Dunn’s test) of percent changes of rectal volumes, fractions vs planning volume*

| *Region* | *Variable* | *Group* | *Planning* | *Fractions* | *p-value^1^* | *Effect Size* | *Kruskall ^2^* |
| --- | --- | --- | --- | --- | --- | --- | --- |
| Anorectum | V30Gy (%) | 1 | 7.7 (4.8 – 10.5) | 7.5 (6 – 8.9) | 1.000 | 0.0000000 | 0.481 |
|  |  | 2 | 8.7 (6.3 – 11.2) | 7.4 (5.9 – 8.8) | 0.616 | -0.1428571 |  |
|  |  | 3 | 7.1 (5.3 – 8.8) | 7.6 (6.3 – 8.8) | 0.490 | 0.1428571 |  |
|  |  | 4 | 9 (7.6 – 10.4) | 10.5 (8.5 – 12.4) | 0.060 | 0.4285714 |  |
| Anorectum | V20Gy (%) | 1 | 13.1 (8.1 – 18.2) | 13.2 (11.3 – 15.1) | 0.950 | -0.1428571 | 0.782 |
|  |  | 2 | 17.1 (12.8 – 21.4) | 19.2 (16.8 – 21.6) | 0.950 | -0.1428571 |  |
|  |  | 3 | 13.7 (10.9 – 16.6) | 15 (13.1 – 16.9) | 0.379 | 0.2857143 |  |
|  |  | 4 | 16.6 (14.6 – 18.6) | 18.3 (15.3 – 21.3) | 0.315 | 0.1428571 |  |
| Anorectum | Maximum (Gy) | 1 | 37.8 (37.7 – 38) | 39 (38.5 – 39.4) | 0.001 | 0.8571429 | 0.259 |
|  |  | 2 | 38.1 (37.8 – 38.3) | 38.8 (38.4 – 39.3) | 0.006 | 0.5384615 |  |
|  |  | 3 | 38.1 (37.8 – 38.3) | 39.9 (39.2 – 40.6) | 0.005 | 0.7142857 |  |
|  |  | 4 | 37.9 (37.8 – 38.1) | 39.8 (39.3 – 40.3) | 0.001 | 1.0000000 |  |
| Anorectal Wall | V28Gy (%) | 1 | 14 (11.7 – 16.3) | 12.3 (10.4 – 14.2) | 0.414 | -0.2857143 | 0.325 |
|  |  | 2 | 13.9 (10.3 – 17.6) | 12.7 (10.8 – 14.6) | 0.802 | 0.1428571 |  |
|  |  | 3 | 9.1 (6.5 – 11.8) | 14.7 (13.8 – 15.7) | 0.079 | 0.1428571 |  |
|  |  | 4 | 14 (9 – 19) | 15.7 (13.1 – 18.2) | 0.851 | -0.1428571 |  |
| Anorectal Wall | V18.5Gy (%) | 1 | 18.6 (15 – 22.2) | 20 (17.5 – 22.6) | 0.572 | -0.2857143 | 0.259 |
|  |  | 2 | 21.3 (16.3 – 26.2) | 21.9 (20.2 – 23.6) | 0.286 | 0.2857143 |  |
|  |  | 3 | 15.5 (12.3 – 18.7) | 21 (18.8 – 23.2) | 0.079 | 0.2857143 |  |
|  |  | 4 | 21.1 (15.5 – 26.8) | 24.4 (21.3 – 27.6) | 0.802 | -0.1428571 |  |
| Anorectal Wall | Maximum (Gy) | 1 | 37.8 (37.7 – 38) | 38.8 (38.4 – 39.2) | 0.003 | 0.7142857 | 0.069 |
|  |  | 2 | 37.9 (37.7 – 38) | 38.3 (37.9 – 38.7) | 0.013 | 0.6666667 |  |
|  |  | 3 | 37.8 (37.6 – 38.1) | 38.2 (37.5 – 39) | 0.033 | 0.4285714 |  |
|  |  | 4 | 37.8 (37.7 – 37.9) | 39.6 (39.1 – 40.1) | 0.001 | 1.0000000 |  |
| Anal Canal | Mean (Gy) | 1 | 9.1 (4.8 – 13.4) | 7.7 (7.4 – 8.1) | 0.490 | 0.1428571 | 0.114 |
|  |  | 2 | 11.1 (8.7 – 13.6) | 10.9 (10.6 – 11.2) | 0.124 | -0.3846154 |  |
|  |  | 3 | 9 (6.5 – 11.5) | 8.7 (8.5 – 8.9) | 0.209 | -0.1428571 |  |
|  |  | 4 | 12.6 (9.9 – 15.4) | 13.1 (12.3 – 14) | 0.149 | 0.1428571 |  |
| Anal Canal | V25cc (%) | 1 | 1.4 (-0.2 – 2.9) | 0.6 (0.4 – 0.7) | 0.900 | -0.1428571 | 0.000 |
|  |  | 2 | 1.9 (1.2 – 2.6) | 1.1 (0.9 – 1.3) | 0.006 | -0.8571429 |  |
|  |  | 3 | 1.7 (0.4 – 2.9) | 1.4 (1.3 – 1.6) | 0.124 | -0.3846154 |  |
|  |  | 4 | 2.2 (1 – 3.5) | 3.2 (2.8 – 3.5) | 0.005 | 0.7142857 |  |
| Anal Canal | Maximum (Gy) | 1 | 37.5 (36.6 – 38.3) | 37.7 (36.9 – 38.5) | 1.000 | 0.2307692 | 0.000 |
|  |  | 2 | 37.6 (37.3 – 37.9) | 37.6 (37.3 – 37.9) | 0.108 | -0.6666667 |  |
|  |  | 3 | 37.7 (37.2 – 38.2) | 37.7 (37.4 – 38.1) | 0.197 | -0.2727273 |  |
|  |  | 4 | 37.7 (37.5 – 37.8) | 38.5 (38.2 – 38.8) | 0.001 | 1.0000000 |  |

*1 Wilcoxon test for in group comparison of median volumes and dosimetrics*

*2 Kruskall-Wallis for group comparison of percent changes of CT*

*Appendix C. Median values of rectal volumes and dosimetric data of planning CT (plan) compared to treatment fractions (Fract.)*

| **Group A** | **Group B** | **Z-score**  **V25cc** | **p-value**  **V25cc** | **Adjusted p-value**  **V25cc** | **Z-score**  **Maximum** | **p-value**  **Maximum** | **Adjusted p-value**  **Maximum** |
| --- | --- | --- | --- | --- | --- | --- | --- |
| 1 | 2 | -1.44 | 0.15 | 0.90 | -1.59 | 0.11 | 0.67 |
| 1 | 3 | -0.312 | 0.76 | 1 | -1.46 | 0.14 | 0.86 |
| 1 | 4 | 2.69 | 0.00 | 0.04 | 2.10 | 0.03 | 0.21 |
| 2 | 3 | 1.12 | 0.26 | 1 | 0.13 | 0.89 | 1 |
| 2 | 4 | 4.20 | 0.00 | 0.00 | 3.70 | 0.00 | 0.00 |
| 3 | 4 | 3.01 | 0.00 | 0.01 | 3.57 | 0.00 | 0.00 |

*Appendix D: Post hoc comparison (Dunn’s test) of Anal Canal V25cc and maximum*

| **Group** | **Number fractions ≥1.0 mm** | **Total fractions** | **Percentage (%)** |
| --- | --- | --- | --- |
| 1: No preparation | 50 | 69 | 72.5 % |
| 2: Diet + laxatives | 42 | 67 | 62.7 % |
| 3: Laxatives only planning CT | 59 | 70 | 84.3 % |
| 4: Laxatives | 55 | 70 | 78.6 % |
| Chi-square test: χ² = 9.24, df = 3, p = 0.0262 | | | |

*Appendix E: intrafractional motion ≥1.0 mm per treatment group*

|  | **X-displacement** | | | **Y-displacement** | | | **Z-displacement** | | |
| --- | --- | --- | --- | --- | --- | --- | --- | --- | --- |
| **Group** | **Mean** | **\|Mean\|** | **SD** | **Mean** | **\|Mean\|** | **SD** | **Mean** | **\|Mean\|** | **SD** |
| 1: No preparation | -0.05 | 0.75 | 0.96 | 0.05 | 1.26 | 1.94 | 0.48 | 1.61 | 2.23 |
| 2: Diet + Laxatives | -0.01 | 0.50 | 0.68 | 0.77 | 1.23 | 1.92 | 0.95 | 1.31 | 1.52 |
| 3: Laxatives only planning-CT | 0.68 | 1.02 | 1.17 | 0.29 | 1.34 | 1.90 | 1.25 | 1.49 | 1.69 |
| 4: Laxatives | 0.12 | 0.89 | 1.26 | -0.39 | 1.26 | 1.61 | 0.31 | 1.60 | 2.18 |

*Appendix F: Mean displacement of CBCT per group per axis (x, y, z) expressed in mm per group. Mean = directional displacement; |Mean| = Mean of absolute displacements; SD = Standard deviation; x = Left-Right; y = Cranio-Caudal; z = Anterior-Posterior.*

**
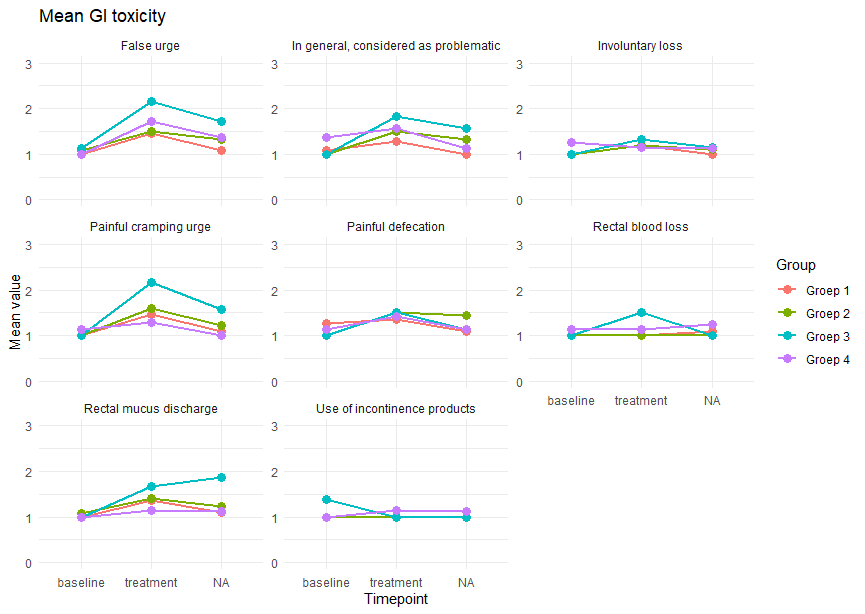
**

*Appendix G: Average Scores per Questionnaire Item Across groups*
